# Knowledge Extraction and Improved Data Fusion for Sales Prediction in Local Agricultural Markets†

**DOI:** 10.3390/s19020286

**Published:** 2019-01-12

**Authors:** Washington R. Padilla, Jesús García, José M. Molina

**Affiliations:** 1 Research Group Ideia Geoca Quito, Salesian Polytechnic University Engineer Systems, Quito 170131, Ecuador; wpadillaa@ups.edu.ec; 2 Applied Artificial Intelligence Group, Carlos III University, Madrid 28270, Spain; molina@ia.uc3m.es

**Keywords:** predictive analysis, data mining, alternative circuits of commercialization, association rules, time series, spatial prediction, kriging and co-kriging

## Abstract

In this paper, a monitoring system of agricultural production is modeled as a Data Fusion System (data from local fairs and meteorological data). The proposal considers the particular information of sales in agricultural markets for knowledge extraction about the associations among them. This association knowledge is employed to improve predictions of sales using a spatial prediction technique, as shown with data collected from local markets of the Andean region of Ecuador. The commercial activity in these markets uses Alternative Marketing Circuits (CIALCO). This market platform establishes a direct relationship between producer and consumer prices and promotes direct commercial interaction among family groups. The problem is presented first as a general fusion problem with a network of spatially distributed heterogeneous data sources, and is then applied to the prediction of products sales based on association rules mined in available sales data. First, transactional data is used as the base to extract the best association rules between products sold in different local markets, knowledge that allows the system to gain a significant improvement in prediction accuracy in the spatial region considered.

## 1. Introduction

Fusion systems allow the integration of heterogeneous sensor data in databases, including knowledge rules, contextual description, external knowledge bases, etc., in order to obtain a general description of real situations. The goal of this process of information fusion is to take the best decisions based on a global view of situations.

The idea behind this work is to find specific patterns in the behavior of consumption of agricultural products: the relationship among the quantities of each product. Information Fusion and Artificial Intelligence (IF/AI) techniques are used to extract association rules for improving the prediction of the consumption of agricultural products. This prediction allows the establishment of better strategies to improve local operations in the network of markets in Ecuador named “CIALCO” (the Spanish acronym for Alternative Circuits of Marketing). This country is crossed by the equator and its territory extends both north and south of latitude zero. This work is centered in the provinces of Tungurahua and Chimborazo. These regions are located in the south and central region of Ecuador, and data were collected on sales of agricultural products in these marketing circuits, created to establish a direct relationship between farmers and consumers. 

The final goal is to increase incomes of the people in the Andean region of Ecuador that works on small farmers, to prevent the migration from this area to larger population centers. There are several markets in the CIALCO circuit; the analysis carried out in this work is related to information of people involved in markets of agricultural fairs. Fairs are places where farmers meet periodically to sell products to consumers and conduct their business [1].

The first source of data was provided by the Ministry of Agriculture and Livestock of Ecuador. The dataset contains weekly performance of sales of products that were sold by small farmers located in the Ecuadorian provinces of Tungurahua and Chimborazo in 2014. The data for each fair is defined by the date, the place, and the volume of sales of products. An average of 300 items per week were sold. This research is based on previous work on information fusion and data mining techniques [2] and the ability to extract knowledge from the fusion of information [3]. Some preliminary results were presented by the same authors at the 2018 International Conference of Information Fusion, Cambridge, UK, on 10–13 July 2018 [4]. In this work, a theoretical approach of fusion system is presented to integrate information from different sources (a net of soft sensors deployed in a certain region) able to improve the predictions of sales by exploiting the association relations mined in the available data and compare their impact with respect to other available magnitudes to fuse such as close population and climatic variables. Associative relations mined from available datasets have proven to be a powerful means to discover causal relations useful for understanding the information coming from heterogeneous sources in different domains such as education [5], smart cities [6], or pervasive computing [7].

In this work, which is focused on the agricultural domain, the composition of several geographically dispersed local markets represents a global market. In each local market, the reported sales are dealt as a soft sensor measuring information about sales, giving information for each product and its associative relationships with the rest. Each market generates the following observational data:

- Local Market Information (LMI): geographical position, name of products, number of sellers, quantity of population that uses the local market, etc.
- For each sale and for each product, the quantity of product acquired in each sale.

The second source of data was provided by the National Institute of Meteorology and Hydrology of Ecuador, where climatic information is recorded for each geographic region based on meteorological sensor sources (temperature, precipitation, humidity, etc.). A third source of data considered in this study was provided by the National Institute of Statistics and Census of Ecuador, where information about the estimated population in each region is stored. At the end, the system is composed of a net of geographically dispersed soft sensors able to obtain data from local markets, fused with hard sensors providing climatic data about each market and also the population of cities close to the market.

The rest of this paper is organized as follows: Section 2 presents an overview of works related to agricultural production. Section 3 proposes a new view of the general problem as a hard and soft information fusion and the algorithm to predict agricultural prediction using special information. Section 4 presents the methodology to integrate data mining to generate relevant knowledge to improve the information fusion process. Section 5 presents the analysis of the target scenario, and Section 6 concludes the paper.

## 2. Related Works

A great effort in sustainable global agricultural production, Sustainable Development Goals from [8], has been carried out in recent years. However, many factors interact, such as extreme weather patterns, rising levels of population and wealth, water scarcity, increases in energy costs, and civil conflicts. Two complementary perspectives have been used to better prepare for disruptions in food supply and global crop market price fluctuations, helping to reduce global food insecurity:

(1) Monitoring system will be able to guarantee timely and accurate information on current food production;
(2) More accurate forecasting techniques for better understanding the key risks in food supply

There are many agricultural monitoring systems on a regional and national level. The main systems analyzed in [9] are:

- The Global Information and Early Warning System (GIEWS) from the Food and Agriculture Organization (FAO) of the United Nations.
- The Famine Early Warning Systems Network (FEWSNET) from the United States Agency for International Development (USAID).
- The Monitoring Agriculture with Remote Sensing (MARS) system from the European Commission (EC).
- CropWatch in China.
- The Crop Explorer service, which provides remote-sensing-based information used by agricultural economists and researchers to predict global crop production.
- GEOGLAM6, a flagship initiative from GEO (Group on Earth Observations), which was endorsed by the G20 in 2011.
- Seasonal Monitor System of the World Food Programme (WFP).
- Anomaly Hot Spots of Agricultural Production (ASAP)9, launched by the Joint Research Centre (JRC) of the European Commission (EC) in June 2017.

Several models and algorithms have been developed to predict the yield of agricultural productions. Prediction uses soil properties and environmental conditions as data to find correlations using several techniques:

- Linear correlation of yield with soil properties and environmental conditions [10];
- Linear methods, especially multiple linear regressions, to predict yields using soil properties [11];
- Nonlinear methods (Artificial Neural Networks- and fuzzy logics) for yield prediction [12,13].

In general, in these kinds of studies, authors work with different factors, mainly soil properties and farm inputs. Several uncontrolled factors could affect agricultural production; therefore, even complex and mathematical models cannot give accurate results. One of the main factors is related to climate variability [14].

In this work, a regional monitoring system of agricultural productions has been developed as a decentralized data fusion process. The main differences with respect to these previous cited works are:

(1) In previous works, authors have tried to forecast the production of some specific products and tried to correlate the amount of production with external conditions. In this paper, we work with several products that could be correlated.
(2) In this work, the monitoring system is modeled as a Data Fusion System, where information from several farms is aligned in time and space and then fused to obtain a global view of production.
(3) Besides an analysis of contextual information (meteorological and close population), a measure of the improvement of using this information is included.
(4) In this work, a “Monitoring Agriculture Fairs System” is proposed which systematically analyzes the relationships among variables based on sales (transactions) in order to improve the accuracy of prediction models for agricultural production.

## 3. Spatial Predictions Based on Data Fusion 

In general, sensors are defined as sources of information from the environment. In real environments, sensors are composed of specific hardware and software dedicated to taking measurements of physical variables (such as location, size, temperature, pressure, etc.). In virtual environments, such as social networks, webs, etc., sensors are specific software tools that extract information from the digital world. These two different types of sensors can be labeled as hardware sensors (h) and software sensors (s). Both types of sources generate information about the environment, and this definition allows to distinguish between real and digital environments [15]. Hard/soft data fusion has been shown to be an effective approach to improve models and understand situations in different domains. For instance, the work in [6] deals with the texts posted from persons as a distributed social sensor system which can be correlated with physical sensor data (audio, temperature, pollution, etc.) to improve information available for services provided in smart cities. 

Therefore, a sensor can be defined as a general data source in the following way:

(1) $$S_{i}^{t}:\;\mathrm{Sensor}\;i\;\mathrm{of}\;\mathrm{type}\;t,\;\mathrm{with}\;t\in \left\{h,s\right\}$$

Besides, several sensors of diverse types could be placed together in a platform of sensors to take advantage of measuring several variables in a coordinated way. For example, a meteorological station is composed of several hardware sensors (temperature, atmospheric pressure, altitude, etc.) and may include software sensors (human observations and forecast reports), or a surveillance system could be composed of a set of hard sensors (radar, camera and infrared camera aligned to detect any object in the search field) and may include human inputs from human operators.

A sensor platform could be defined in the following way:

(2) $$P_{j}={\left\{S_{ij}^{t}\right\}}_{i=1}^{n}:\;\mathrm{Platform}\;j\;\mathrm{composed}\;\mathrm{of}\;n\;\mathrm{sensors}\;\mathrm{of}\;\mathrm{different}\;\mathrm{types}.$$

**Figure 1:**
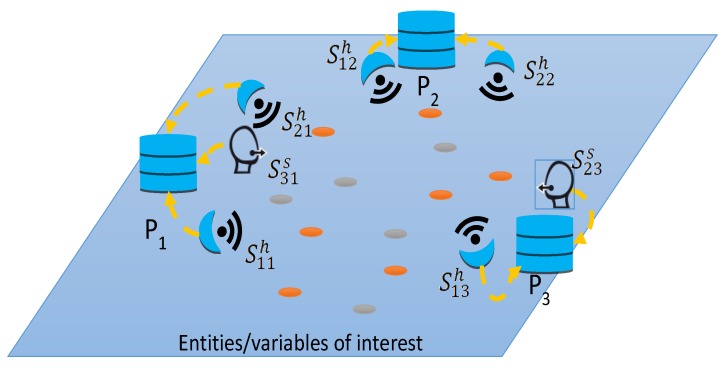
A deployed network including hard and soft sensors in platforms *P_1_*, *P_2_*, and *P_3_*.

In general, platforms should be distributed in the environment to cover a large area, and, in many cases, coverage areas are overlapped to improve detections in the boundaries, as shown in Figure 1. The information captured by platforms is sent to a fusion centre where it is integrated using spatial and temporal information.

The fused information is used to predict future situations. This prediction depends on a model that could be based on measurements from a single source, for example current and past positions of a target to predict future positions based on measures and the movement model. In some situations, the information from the set of sensors of the platform could be integrated, and the complementary information of each one allows the final prediction to be improved. For example, the radar position, the visual position, and the thermic position could be combined to generate a “best” position to improve the prediction of future position, or the predicted future position based on radar could be improved considering the actual video position. 

In the fusion centre, information of the network is received, and measures of each sensor are stored to improve the final prediction:

(3) $$I_{ij}^{t}:\;\mathrm{Information}\;\mathrm{from}\;\mathrm{Sensor}\;i\;\mathrm{of}\;\mathrm{platform}\;j\;\mathrm{of}\;\mathrm{type}\;t.$$

That could be seen as a sequence of values registered in a database, defining each register as a vector with the corresponding values (time stamp, platform *j*, sensor *i*, type of sensor *t*, value). These vectors would be stored in a database, as shown in Table 1.

The final prediction could be based on the information of only one sensor of the platform, for example, tracking a target using the radar of one platform. In this problem, we have a primary source, that is composed of a deployment with platforms of soft sensors located in each local market. Each platform is defined by the market data: position, number of products, number of sellers, quantity of population that uses the local market, etc., and has a set of soft sensors able to measure the level of sales of each product:

(4) $$I_{ij}^{t}:\;\mathrm{Information}\;\mathrm{of}\;\mathrm{level}\;\mathrm{of}\;\mathrm{sales}\;\mathrm{of}\;\mathrm{product}\;i\;\mathrm{from}\;\mathrm{market}\;j\;\mathrm{of}\;\mathrm{type}\;t.$$

Each platform is composed of two other soft sensors:

- A soft sensor able to measure the climatic floors for each geographic region where the market is located. This is a software process applied over data coming from hard sensors (pressure, humidity, luminosity, etc.):
  (5) $$I_{ij}^{t}:\;\mathrm{Information}\;\mathrm{of}\;\mathrm{climatic}\;\mathrm{characteristic}$$
  *i*-th source from market *j* of type *t* (e.g., for temperature, humidity, etc.)
- A soft sensor able to measure the population in each one of the cantons of the study.

$I_{ij}^{t}$: Information of population characteristic i from market j of type t (percentage, absolute, etc.)

Considering this spatially distributed data fusion problem, future predictions could be computed exploiting the geo-statistical properties of variables. The prediction of a variable spatially distributed is a specific problem considering local information. Citing [16], “in the geographical space everything is related to everything, but the closest spaces are more related to each other”. This process usually starts by defining the function taking values on a certain spatial region D. This function provides a set of random variables for x taking values in the domain D, being defined as *Z = {Z(x)*, *x*∈D}. The values are also random, being the expectation and variance, first and second order moments, defined as usual:

*m*(*x*) = *E*[*Z*(*x*)] (6)

(7) $$\sigma^{2}(x)\;=\;\mathrm{var}[Z(x)]\;=\;E\{{\left[Z(x)-m(x)\right]}^{2}\}\;=\;E[Z{\left(x\right)}^{2}]-m(x{)}^{2}$$

This last value assesses the scatteredness of *Z(x)* around the expectation, while the covariance between different points in D is defined as:

(8) $$C\left(x_{1},x_{2}\right)=E\left[Z\left(x_{1}\right)Z\left(x_{2}\right)\right]-m\left(x_{1}\right)m\left(x_{2}\right)$$

This value characterizes the interaction between $Z\left(x_{1}\right)$ and $Z\left(x_{2}\right)$, usually integrated in the semi variogram function, defined as:

(9) $$\gamma \left(x_{1},x_{2}\right)\;=\;\frac{1}{2}var\left[Z\left(x_{1}\right)-Z\left(x_{2}\right)\right]$$

which reflects the way in which a point has influence on another point at different distances. The relation between variogram and covariance is given by:

(10) γ(h)=C(0)−C(h)

As defined above, given a set of *Z* values provided by sensors deployed in n certain sites, {*x_1_*, …, *x_n_*}, the variogram can be estimated considering the separation vector h as:

(11) $$\gamma \left(h\right)=\frac{1}{2}E\left\{{\left[Z\left(x+h\right)-Z\left(x\right)\right]}^{2}\right\}$$

In the case that γ(h) is isotropic (identical in all directions of the space), it does not depend on the angle of vector h, but only on its magnitude, |*h*|.

In the general case, it is difficult to obtain the experimental variogram from data, given the scarce distances and directions, and usually a theoretical model must be adjusted with the available dataset. A quite generic model for variogram considers growing from the origin until a distance for stabilization, around a plateau, so that the random variables *Z(x)* and *Z(x + h)* are correlated when the length of the separation vector h is lower than a certain distance, the zone of influence, and beyond |*h*|= a the variogram keeps constant (the plateau). For instance, a spherical variogram of reach a and plateau C is defined as [17]:

(12) $$\gamma \left(h\right)=|\begin{array}{c} C\left\{\frac{3}{2}\frac{\left|h\right|}{a}-\frac{1}{2}{\left(\frac{\left|h\right|}{a}\right)}^{3}\right\}\;if\;\left|h\right|\;\leq a\\ C\;\;otherwise \end{array}$$

In the isotropic case, assuming independent direction of the semi variance, the vector h is replaced with its magnitude, ‖*h*‖. In this case, the variogram is computed taking pairs of data $Z\left(s_{i}\right),\;Z\left(s_{i}+h\right)$ along the available distances in interval $\bar{h_{j}}$, defined as:

(13) $$\hat{\gamma }\left(\bar{h_{j}}\right)=\frac{1}{2N\left(h\right)}\sum_{i=1}^{N_{h}}{\left(Z\left(s_{i}\right)-Z\left(s_{i}+h\right)\right)}^{2},\;\forall h\;\in \;\bar{h_{j}}$$

In some cases, the experimental variogram shows changes of slope in certain distances. In these cases, the variogram model can be a sum of simple models (nested structures):

(14) $$\gamma \left(h\right)=\gamma_{1}\left(h\right)+\gamma_{2}\left(h\right)+\ldots +\gamma_{s}\left(h\right)$$

In these cases, the adjustment is not based only on experimental data, but it must consider also contextual information about the region. More details appear in [18].

Kriging is a linear prediction model corresponding to the unbiased linear estimator. There are different types of kriging depending on the average of population: ordinary and simple.

In ordinary kriging, a stationary *Z* random function is obtained:

(15) $$\{\begin{array}{c} \forall x\;\in V,\;E\left[Z\left(x\right)\right]=m\;\;unknown\;\;\;\;\;\;\;\;\;\;\;\;\;\;\;\;\;\;\;\;\\ \forall x,x+h\;\in V,\;cov\left[Z\left(x+h\right),Z\left(x\right)\right]=C\left(h\right) \end{array}$$

where *V* is the neighborhood considered in the process. The model is defined as:

(16) $$Z^{*}\left(x_{0}\right)=\;a+\sum_{\alpha =1}^{n}\lambda_{\alpha }Z\left(x_{\alpha }\right)$$

where $x_{0}$ is the place to predict the variable, {$x_{\alpha }$, α = 1,…, *n*} are the available sites with training data, and {$\lambda_{\alpha },\;\alpha =1,\ldots ,n$} are the weights to be computed, together with constant a. A first constraint of estimator is to be unbiased, (mean value of error must be zero):

(17) $$E\left[Z^{*}\left(x_{0}\right)-Z\left(x_{0}\right)\right]=0\;=a+\overset{n}{\underset{\alpha =1}{\sum }}\lambda_{\alpha }E\left[Z\left(x_{\alpha }\right)]-E[Z\left(x_{0}\right)\right]=a+m\left(\overset{n}{\underset{\alpha =1}{\sum }}\lambda_{\alpha }-1\right)$$

With this constraint, the weights to minimize the variance of estimator can be computed as:

(18) $$minimize\;\left(var\left[Z^{*}\left(x_{0}\right)-Z\left(x_{0}\right)\right]\right)\;\;=\sum_{\alpha =1}^{n}\overset{n}{\underset{\beta =1}{\sum }}\lambda_{\alpha }\lambda_{\beta }C\left(x_{\alpha }-x_{\beta }\right)+C\left(0\right)-2\sum_{\alpha =1}^{n}\lambda_{\alpha }C\left(x_{\alpha }-x_{\beta }\right)$$

Replacing the variogram by the covariance through the relationship γ(h)=C(0)−C(h), the kriging prediction is obtained as follows:

(19) $$\{\begin{array}{c} \overset{n}{\underset{\beta =1}{\sum }}\lambda_{\beta }\gamma \left(x_{\alpha }-x_{\beta }\right)-\mu =\gamma \left(x_{\alpha }-x_{0}\right)\;\;\forall \alpha =1\ldots n\\ \overset{n}{\underset{\alpha =1}{\sum }}\lambda_{\alpha }=1 \end{array}$$

The core of the fusion process is carried out with co-kriging, a technique using multiple spatial variables to build an extended prediction model. This multivariable kriging process takes as input a set of m available spatial variables, $\left\{Z_{1},\ldots ,Z_{m}\right\}$, previously aligned with the variable to predict and collocated in the same coordinates. The prediction equation is extended for this case as follows:

(20) $$Z_{i}{}^{*}\left(x_{0}\right)=a+\sum_{\alpha =1}^{n}\lambda_{\alpha }Z_{i}\left(x_{\alpha }\right)+\sum_{j=1,j\neq i}^{m}\sum_{\alpha =1}^{n}\lambda_{\alpha j}Z_{j}\left(x_{\alpha }\right)$$

It is an estimation method that minimizes the error variance by exploiting the cross-correlation between the available variables. In an analogous way, the error covariance of prediction $Z_{i}{}^{*}\left(x_{0}\right)$ can be expressed as a function of the own coefficients, $\lambda_{\alpha },$ and the coefficients for the collocated variables, $\left\{\lambda_{\alpha j}\right\},\;j=1,\ldots n,\;j\neq i$. Consequently, the expression to minimize, analogous to Equation (17), will depend both on the own variogram, $\gamma_{i}\left(h\right),$ and on the crossed variograms among the variables,$\;\gamma_{ij}\left(h\right)$. 

Therefore, the first step of co-kriging also consists in computing the multivariable variogram in order to estimate the semi variances among all variables:

(21) $$\gamma_{ij}\left(h\right)=\frac{1}{2}E\left\{\left(Z_{i}\left(s\right)-Z_{i}\left(s+h\right)\right)\left(Z_{j}\left(s\right)-Z_{j}\left(s+h\right)\right)\right\}$$

This crossed variogram between variables $Z_{i},$ $Z_{j}$ can be estimated with the available training data:

(22) $${\hat{\gamma }}_{ij}\left(h\right)=\frac{1}{2|N\left(h\right)|}\sum_{N\left(h\right)}\left[Z_{i}\left(x_{\alpha }\right)-Z_{i}\left(x_{\beta }\right)\right]\left[Z_{j}\left(x_{\alpha }\right)-Z_{j}\left(x_{\beta }\right)\right]$$

where the set *N(h)* is defined as {α,$\;\beta ,\;\mathrm{such}\;\mathrm{that}\;x_{\alpha }-x_{\beta }=h\}$, with variables $Z_{i\;}and\;Z_{j}$ taking values in the corresponding locations $x_{\alpha }\;\mathrm{and}\;x_{\beta }$.

In this way, a methodology for data integration suggested by Doligez et al. [19] is stepwise, progressively integrating data at different scales to improve the interpolation, illustrated in fusing seismic and well data. This stepwise approach can be used to integrate many data types. Once correlations among variables are analyzed, regression or geostatistical methods are used to integrate the data with the co-kriging approach. On the other hand, hybrid models mixing machine learning and geostatistics, such as Neural Network Residual Kriging/Co-kriging (NNRK/NNRCK) [20], have proven their efficiency in real-world mapping problems. 

Building extended models combining individual factors to fit the structure of the data faces the complexity of dimensionality and the risk of oversimplifying the unknown causal relationships of the multiple factors. Usually, it is best to separate the spatial processes whenever possible and try to understand the causal relationships among the available variables. Therefore, a refined fusion strategy is needed to overcome problems of dimensionality or stationarity assumed in statistical methods, or the interpretability problem of machine learning approaches. In this work, the underlying hypothesis is that the most relevant associative patterns can be mined in transactional data and then exploited to improve the efficiency of multivariable models integrating correlated variables. In general, results must be cross-validated to demonstrate the contribution of this approach. Cross validation in geospatial data implies systematically removing points from the data set and re-estimating the predictions based on the model assessed. This will be the means to validate and assess the contribution of the secondary information resulting from the fusion of available data.

## 4. Methodology Based on Data Mining to Improve the Fusion Process

**Figure 2:**
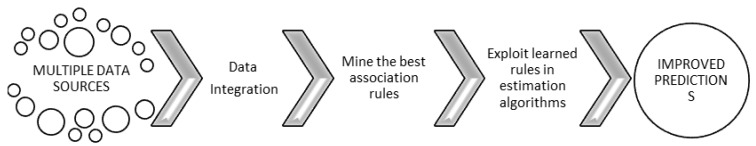
Global process description.

The methodology follows three steps: Fusion, Machine Learning, and Prediction. The Fusion System integrates data available from different platforms (local markets) that contain several data sources (market sales, climatic variables, population data, etc.). As indicated, the system can learn relationships between variables that may be useful for the prediction of future sales based on information considering spatial distributions, meteorological information, etc. An overview of the general process is detailed in Figure 2 and explained below:

(1) Clean and Transform Information
  1.1. Records with no values are deleted
  1.2. Similar values on records are standardized
  1.3. Units are defined for all measurements
  1.4. The set of products are selected for the study
  1.5. Generate spatial structures:
    1.5.1. Create common spatial structures (grids for extrapolation)
    1.5.2. Transform data to a common coordinate system (the same reference for all sources)
  1.6. Final database is generated and prepared for pattern search
(2) Extract best association rules
  2.1. The database of sold products is discretized from transactional data
  2.2. Association algorithms are applied to mine the best association rules
  2.3. Establish the set of products with strongest associations
(3) Estimate future predictions applying geostatistical fusion techniques and validate the hypothesis of strongest conditional dependencies found by data association mining
  3.1. Comparison of the fusion results using the set of most associated variables
  3.2. Comparison of the results using climatic floors
  3.3. Comparison of the results using population
  3.4. Comparison of the results using the rest of the variables
(4) Finally, the improvement of the proposal is analyzed by comparing predictions with a single product vs the data fusion results (using residuals as evaluation metrics with Leave-one-out cross-validation, LOOCV)

In Figure 2, global process for improving predictions is schematized. The first step is the integration of information received from knowledge sources (every local market) generating global information with every available source. From this information, system obtains the best association rules that should be used in the next step to improve predictions of sales (this is the learned sales model that represent the relation among products). The result of the first step is the fusion of local information received from local markets to generate a global view of sales in a region. In the second step, knowledge is extracted from this global view obtained relevant relationships among products. The third step, the final step, applies this knowledge to improve the future prediction of sales. 

The global fusion process system needs knowledge extracted from local markets. In this sense, at the beginning of the process, it gathers representative information after applying the machine learning procedure to extract sales knowledge, and then this learned model is used to generate improved predictions by fusing observations and learned model.

Mining association rules is the way to find causal relations among variables (in the simple way is a rule that find relations between two variables). These rules allow the prediction of changes in the value of a variable based on knowledge of another one. The form of an association rule is {*A*} ⇒ {*B*}—this rule means: “if *A* appears in the register then *B* also should appear in the same register”. This kind of rule is useful to identify relationships between categorical attributes that are not explicit. As in any rule system, set *A* is named antecedent of the rule and *B* is named consequent. Each association rule should be evaluated to assess each quality, and evaluation is based on three common metrics: support, confidence, and lift [21]:

- *Support* (of a rule) is evaluated as the number of instances (register in the data set) the rule covers related to the whole set (of registers in the dataset).
  (23) $$\sup_{a}\left(x\right)=\left|x\right|,{\;\sup}_{r}\left(x\right)=\frac{\left|x\right|}{\left|D\right|},$$
  where *D* is the total set of transactions.

If antecedent *A* and consequent *B* are considered, the support is the intersection set:

sup_a_(*A*⇒*B*) = sup_a_(*A*⋂*B*) (24)

- *Confidence* (of a rule) is evaluated as the number (percentage) of times that consequent B appears among the instances that are selected by the antecedent A. The meaning of this concept is the accuracy of its prediction; it is defined as:
  (25) $$\mathrm{conf}\left(A\Rightarrow B\right)=\frac{sup_{a}\left(A\Rightarrow B\right)}{sup_{a}\left(A\right)}=\frac{\left|A⋂B\right|}{\left|A\right|}.$$
- *Lift* (of a rule) is evaluated as the ratio of observed support considering that A and B were independent:
  (26) $$\mathrm{lift}=\frac{\sup\left(A\Rightarrow B\right)}{\sup\left(A\right)\;x\;\sup\left(B\right)},$$

There are several algorithms that extract association rule from a database. The most representative algorithm for this task is the Apriori algorithm [22]. Explanations about this algorithm in [23,24] clarify that Apriori algorithm finds trends using performance parameters (support, confidence, and lift) evaluated on “a priori” frequent sets (prior knowledge). The algorithm is composed of the following steps:

(1) Generate all item sets *L* with a single element; this set is used to form a new set with two, three, or more elements. All possible pairs are taken so that their *support* equals *minsup*
(2) For every frequent item set *L’* found:

For each subset *J*, of *L’*

Determine all association rules of the form:
If *L’*-*J→J*
Select those rules whose *confidence* is greater than or equal to *minconf*

Repeat **1**, including next element into *L*

As explained, all item sets that satisfy a threshold of minimum support are searched. However, looking for all subsets would not be possible for the exponential size of search space of potential item sets to analyze. The Apriori algorithm prunes candidates with an infrequent subset before counting their supports. This is a Bread-First Search (BFS) process; it ensures that the support values of all subsets of a candidate are known in advance. All candidates of a cardinality k are counted in each scan in order to prune the branches below the support threshold, and then the search descends along the rest in the tree. 

A possible alternative approach could be using Depth-First Search (DFS), expanding the candidate sets from the item sets of one of the nodes of the tree. Obviously, scanning the database for every node would result in tremendous overhead, so counting occurrences in a DFS mechanism is not practical. A more recent approach, called FP-growth, has been introduced in [25], and was shown to be more efficient that Apriori in representative situations [26]. In a preprocessing step, FP-growth builds a condensed representation of the transaction data, called FP-tree. FP-growth does not explore all the nodes of the tree, but directly descends to some part of the item sets in the search space and, in a second step, uses the FP-tree to derive the support values of frequent item set. 

Besides, other recent extensions of Apriori go in the direction of temporal patterns. This algorithm cannot be used in many applications where patterns vary with time. In this case, entities follow periodic patterns such as transportation on time, load with time constraints, some trajectories, etc., and this kind of problem is not considered by Apriori basic algorithm. This kind of problem should be formulated as discovering patterns from dataset considering temporal attributes and try to model how they vary with time. Many algorithms for finding temporal patterns in sequence databases are listed in the bibliography; these algorithms are usually based on sequence mining techniques (or frequent patterns search) and, at the same time, temporal data association. 

There are some extensions of the Apriori algorithm that consider lists of ordered objects using time as items. Then, searched result is the associations of items in the form of sequences of items. Some examples of these algorithms derived from Apriori are Generalized Sequential Pattern (GSP) for spatiotemporal associations [27], Sequential PAttern Discovery using Equivalence classes (SPADE) [28], and Sequential PAttern Mining (SPAM) [29].

Other techniques extract meta-rules describing how relationships vary in time [24,30]. These techniques are also based on APriori mining schema extended to consider time meta-relationships. Some studies conducted in several domains of science have tried to use rules extracted with the Apriori algorithm as a criterion to generate future estimation using associations. Typical works are [31], where the authors analyze the stock of a supermarket, and [32], where authors predict admission decisions by students. Additionally, works such as [33] have searched relationships between extracted rules (association rules) and other techniques such as fuzzy classification.

## 5. Case Study

As mentioned in the introduction, the global market is composed of several geographically dispersed local markets. Each local market provides sales information for each product in the market. Next, we describe first the preparation of data as the first step before fusion and knowledge extraction for sales forecasting.

### 5.1. Data Preparation

The dataset of products that are considered in the analysis is listed in Table 2. In this table, names in Spanish (the original dataset) appear in the first column, their corresponding scientific names appear in the second column, and their corresponding translations into English appear in the third column. The process starts validating the original data provided by the governmental office. Non-significant information is deleted, and product names and units of measure are standardized. The dataset provided contains data stored weekly in local fairs of CIALCO (related to the provinces of Chimborazo and Tungurahua, which belong to the central area of the Andes in Ecuador). The initial dataset is subjected to processing for homogenization data (cleansing data). This cleaning mainly affects the names of products, values for unit sales, and the deletion of products that are not relevant for this work.

The available data has been prepared at two levels in order to apply the methodology described. On the one hand, the sales values reported for each individual product have been aggregated in weeks and locations in order to build the prediction models, and also have been aligned in space and time for fusion in a multivariable model. The tables prepared for each product contain the weekly variation of sales in each spatial location in the 48 weeks of available data.

On the other hand, the original data sheet contains the recorded transactions organized in packages named “canastas” (baskets), each one representing a sale in the local market containing a subset of products in the fair. These are the products contained in each purchase, used to prepare a base table for the search of association rules performed to discover the variables with strongest association and use in the fusion model.

#### 5.1.1. Spatial Data 

Table 2 is complemented in Table 3, with seventh and eighth columns that integrate the X and Y coordinates of each transaction. X-Y coordination is related to the commercialization area that corresponds to the provinces of Tungurahua and Chimborazo in the month of July 2014. X and Y represent the latitude and length of local fairs (a position for each local fair) that participate in this work, respectively. Additionally, a fourth column is integrated corresponding to sales values that reflect the behavior of commercialization of kidney tomato. 

**Figure 3:**
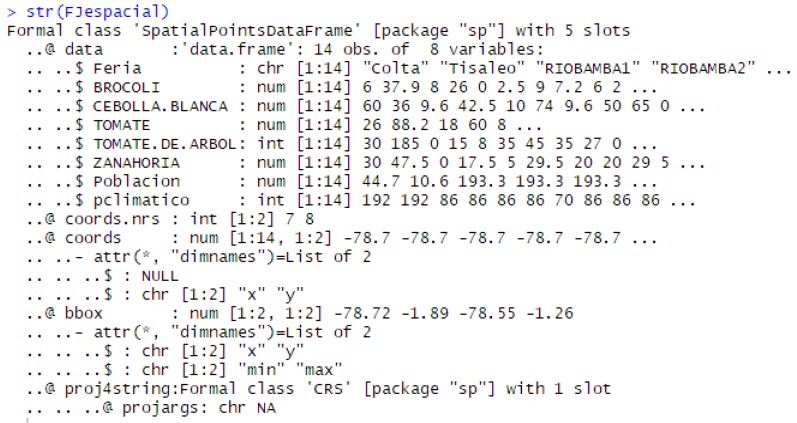
Data structure spatial type.

After this process, the dataset is transformed converting sales data into spatial type structures using latitude and longitude, as shown in Figure 3.

#### 5.1.2. Climatic Zones

**Figure 4:**
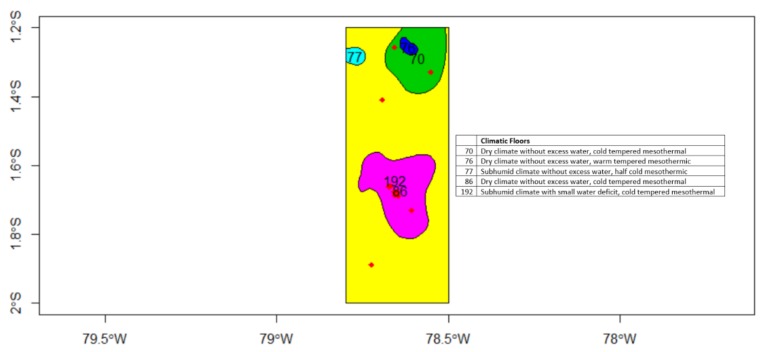
Climatic zones.

The National Institute of Meteorology and Hydrology of Ecuador [34] provides public information related to the climatic floors for several zones in Ecuador (Figure 4). The information provided is a value that corresponds to a different climate category. Each category is associated to a particular interval of variables sensed in the ground network of meteorological stations: humidity, temperature, precipitation rate, etc., so that predefined intervals define each climate category. In the area that corresponds to the 14 fairs, there is a type of mesothermic climate with an average temperature of 18 °C throughout the year, average precipitation of 650 mm, humidity index that is between −16.5 and 10, and potential evaporation with values that vary between 64 and 106 cm.

**Figure 5:**
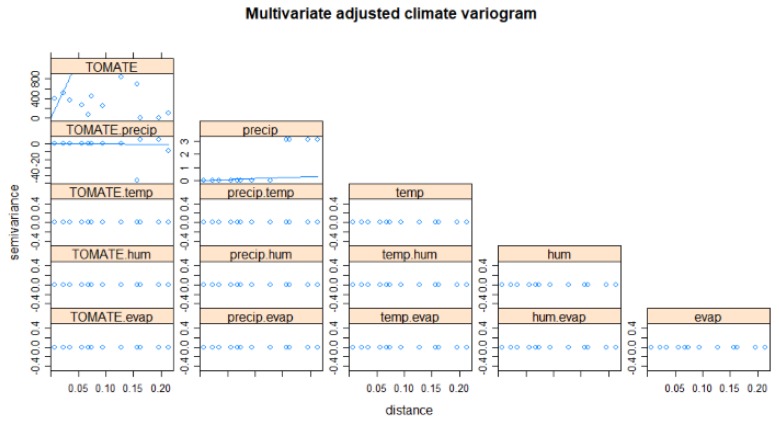
Variograms of climatic variables.

The variation of the climatic variables (Figure 5) does not present a representative slope; the one that contributes for this study is the humidity, with a correlation with the tomato variable.

Another important source of information is population. Population dataset is public information that is provided by the Ecuadorian Institute of Statistics and Censuses. In particular, population information related to the cantons considered in this work can be found in [35]. 

Finally, Table 4 is generated using the information of the presented datasets where coordinates are transformed to common references. Table 4 is a unified file where sales information, population, and climate zones are integrated to build the multivariable prediction model.

### 5.2. Association Rules Mining

As mentioned, the initial file with transactions was analyzed in order to search significant association rules. For each transaction in the available dataset, each agricultural product was checked to determine whether it took part in the sale or not (“t” denotes True and “f” denotes False). However, the negative cases were removed from training set in order to concentrate only on “positive” rules, i.e., relations among products sold together, and avoid rules including absent products in the relationships.

After that, association rules were searched with algorithms available in Weka platform [36]. With a preprocessed training set containing 549 transactions and subsets of 31 items purchased was analyzed to search association rules, setting as parameters a support value greater than or equal to 0.4 (220 transactions in the training set).

**Figure 6:**
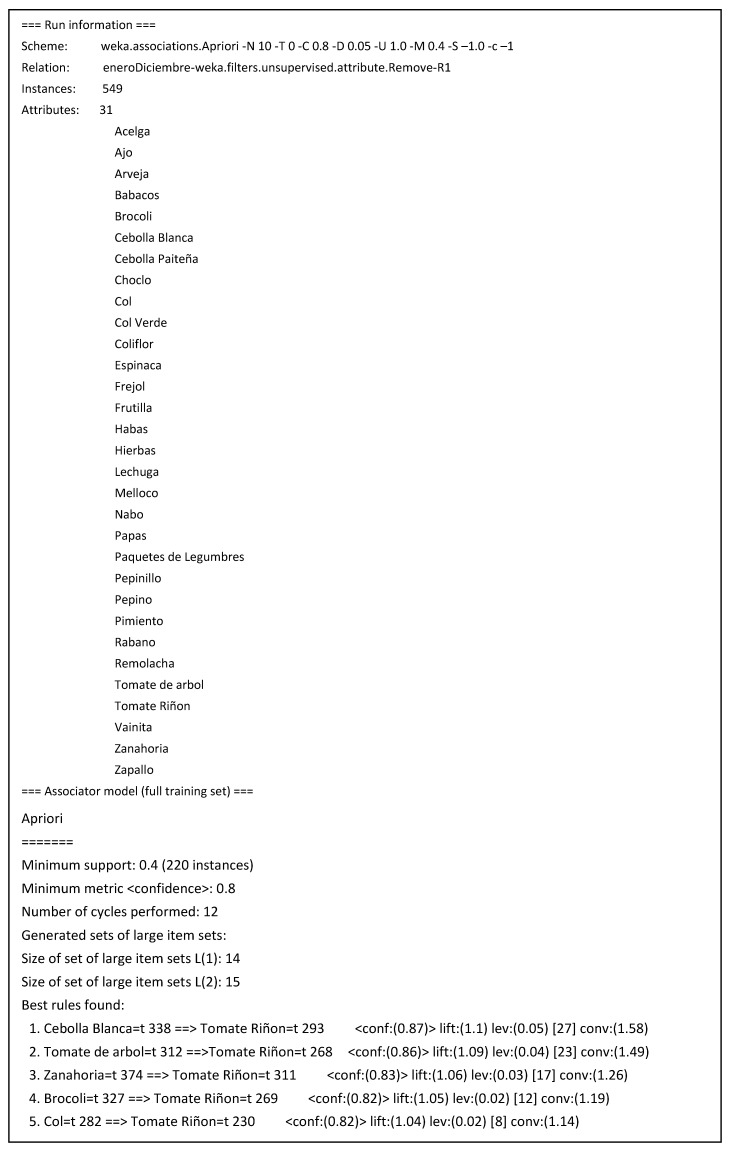
Main association rules found among agricultural products with the Apriori algorithm.

The Apriori algorithm was applied to the dataset containing more than 500 transactions on a weekly basis. It was configured with parameters of minimum support set to 0.4 (220 occurrences) and a confidence value set to 0.8. The main findings were:

- The strongest association was found between white onion and tomato products, with a confidence of 87%. The next strongest were the association rules for tamarillo (86%), carrot (83%), and broccoli (82%). These products were selected as the multivariable set, as indicated in Figure 6.
- The product with the highest commercial ratio of the study sample is tomato.

**Figure 7:**
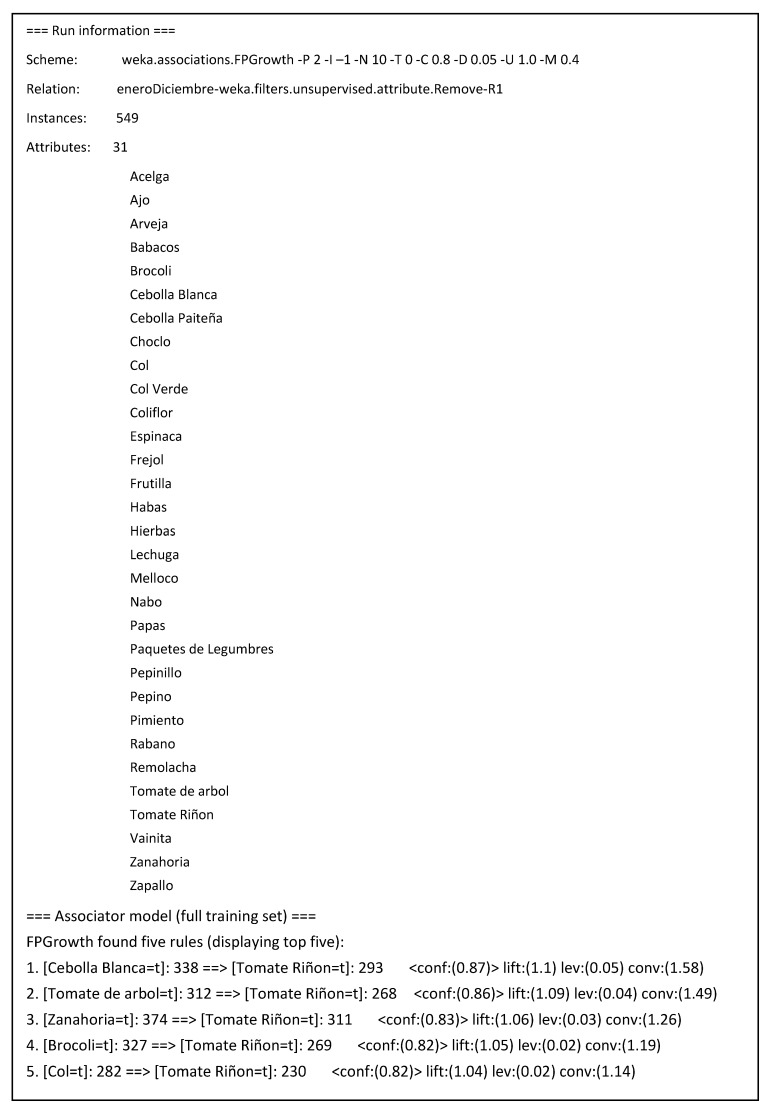
Main association rules found among agricultural products with the FPGrowth algorithm.

As shown in Figure 7, these results were corroborated by applying the FPGrowth algorithm, which found the same five most relevant association rules among agricultural products in the transactions data sheet.

### 5.3. Spatial Prediction 

Spatial prediction was performed first using only the tomato variable as a benchmark, carried out by means of the kriging method with an individual variable. Then, several subsets of the available variables were used in composite structures for a fusion process, based on multivariable co-kriging, in order to perform the predictions and compare with the single-variable situation in order to analyze the relative gain. 

#### 5.3.1. Kriging Prediction

**Figure 8:**
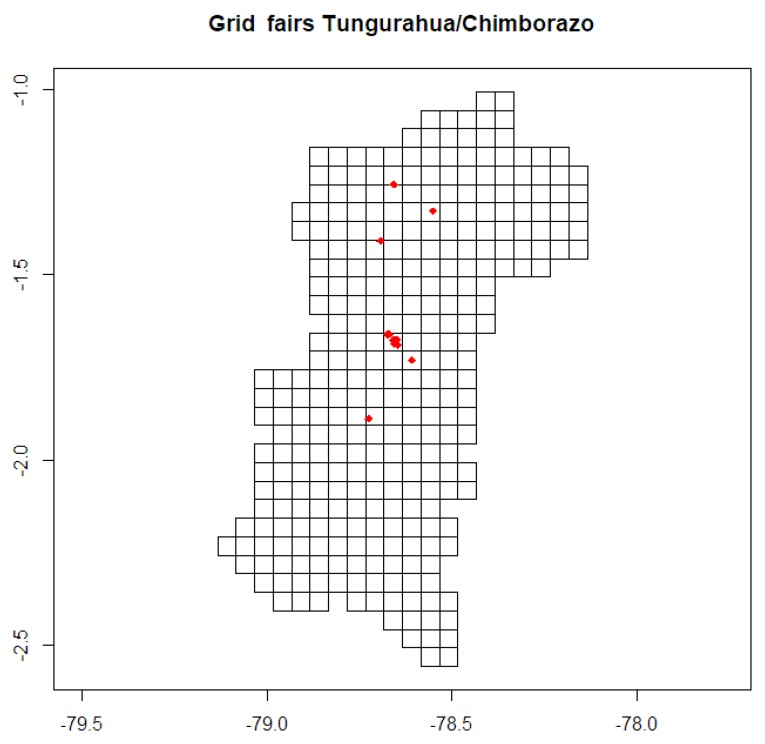
Spatial grid for Tungurahua and Chimborazo provinces.

The available libraries of R studio [37] were applied to process the available spatial data. The first step required is building a grid (or mesh) in order to fix the prediction area, setting certain parameters to describe the structure: cell size set to 0.05, offset = (−79.1085, y = −2.531218), dim x = 21; y = 32. In the central sector of the region, one degree of length is equivalent to 111.32 km, and the distance occupied by the two provinces, in the horizontal interval of 116 km length (−79.133499, −78.0834991) corresponds to 1.049 degrees. Red points in Figure 8 indicate the locations of fairs, and the grid was adjusted to cover the limits of the provinces, with the geographical coordinates (longitude, latitude) indicated in the axes. 

**Figure 9:**
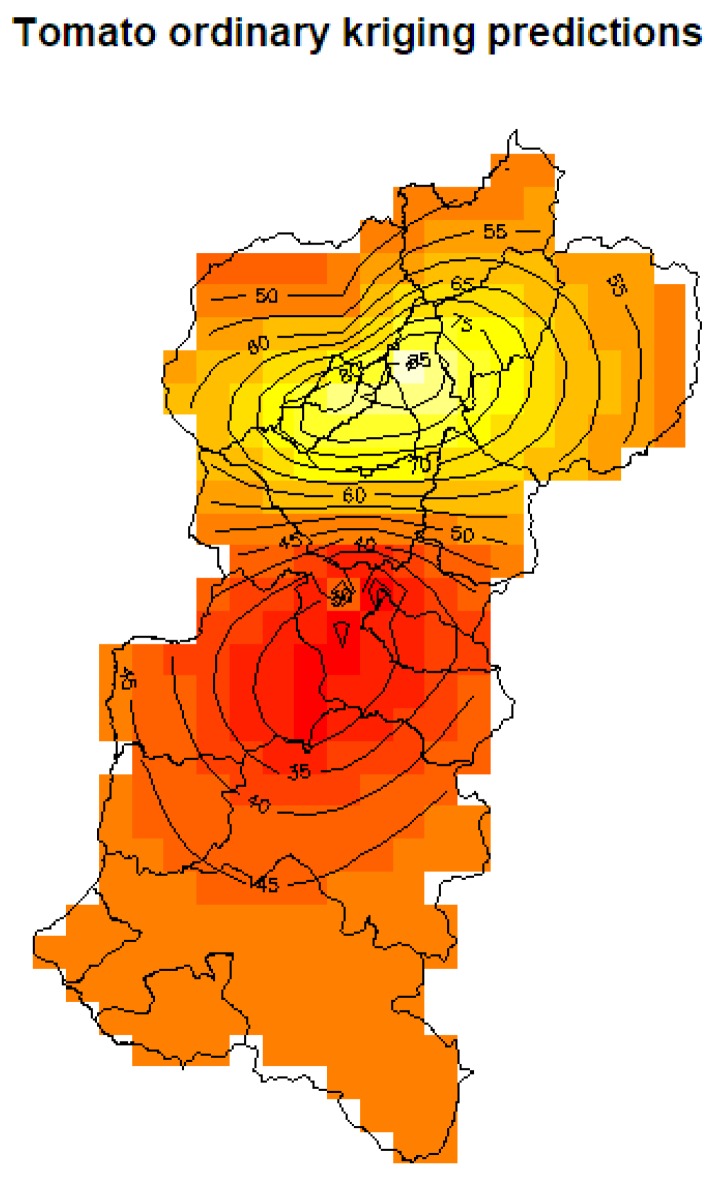
Tomato prediction with ordinary kriging.

The second part consisted of generating the prediction function for the study domain, generated with the function based on the adjustment to the empirical variogram with a model of spherical type. The sale estimate of tomato for the month of July 2014 across the region containing the two provinces considered is shown in Figure 9.

#### 5.3.2. Co-kriging Prediction

**Figure 10:**
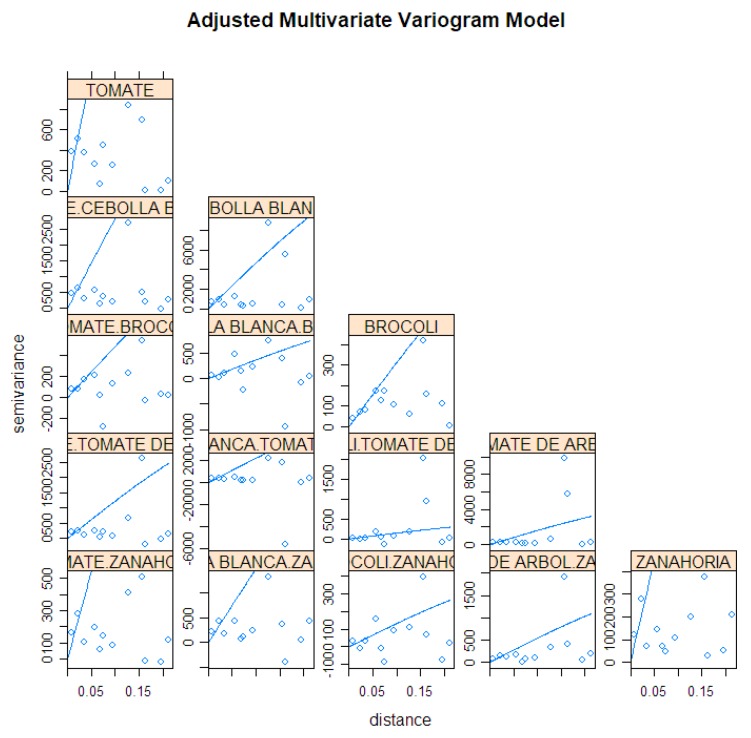
Fitted multivariate variograms of products with strong association.

The prediction of product sales resulting from information fusion was done using the co-kriging method (multivariable kriging) described above, using the available variables aligned in the spatial grid. The correlations and associated variograms are shown in Figure 10, including the variogram models fitted with available data and used in the prediction process. As can be seen, the available data is scarce, so some deviations appear from theoretical model. A full model would require much data to represent all spatial properties in different directions, so the usual solution is a trade-off to fit standard robust models and avoid numerical problems in the spatial regression model.

The analysis of information fusion impact on the results was done by comparing the effects on prediction accuracy, that is, if the errors decrease in predictions. The predictions for tomato sales were computed with a fusion model considering the other variables available in the study, such as population, meteorological variables, as well as the set of products present in the strongest association rules.

LOOCV was applied to compare the different predictions (using the residuals). LOOCV has minimum bias because the training set contains almost the whole dataset. As discussed in [38], LOOCV can achieve the minimum variability both in bias and variance with stable learning schemes as linear regression, and it is usually the selected choice for comparative analysis when the available dataset has limited size.

### 5.4. Discussion of Results

**Figure 11:**
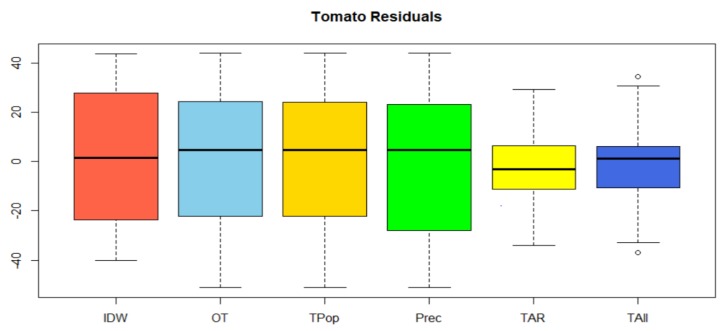
Cross validation of residuals.

Six sets (Figure 11 and Table 5) of data were defined because of future prediction (residuals); these are: inverse distance weighted (IDW) interpolation, which allows us to know a basic value; tomato only (OT), which sets the prediction to future (residual) using kriging techniques; TPop, which is tomato and population with co-kriging; Tomato and Precipitation (Prec), a cokriging technique; TAR, which means tomato association rules; and TAll, which means tomato and all the previous variables. The values of the cross validation for each of the data groups behave similarly within the range of the expected results. We can notice that there is an improvement (residual) between using IDW techniques, calculation with a single variable and multivariate calculation (co-kriging). When presenting the consolidated information, in all cases the fusion of additional information has an impact with respect to the case with a single variable. Including the population and precipitation variables to perform the multivariable future estimate does not decrease the value of the estimated error (residual). Therefore, the fusion of information with the association rules allows a more significant improvement in this prediction process. The fusion of information between the tomato variable and the precipitation (Prec) even presents an increase in the maximum and minimum interval of the residuals. Once the direct relationship is established in the improvement of the future estimation in the marketing of the tomato based on the products resulting from the best association rules (TAR), the data of precipitation and population are added to the information fusion (Tall), establishing an improvement in the value of the average of the residuals.

The decrease in the residual values allows us to infer that the fusion of data from the products with strongest association rules reduces the white noise and improves the predictions of the marketing of the tomato.

## 6. Conclusions

The focus of this paper was centred on the use of transactional information about market sales of small farmers to analyze dependencies among products in order to improve the accuracy of predictions. Dependency was learned from individual sales and recorded and stored in a database to mine association rules that measure the dependency among products. These rules show which products should be considered jointly to predict future values in an extended model. Additionally, contextual information was added and fused with the previous information to analyze the improvement of predictions. The research was focused on a target area for analysis located in two provinces of Ecuador, with available data from alternative marketing circuits.

Besides, a general fusion methodology based on variable selected from association rules mining was applied to improve the predictions. With this improvement in the sales prediction process it would be possible to establish scenarios for consumption maps in order to take strategic decisions aimed at improving the economic income of farmer families. Some limitations have been identified, namely the moderate size of available data and low quality of meteorological variables to integrate, pointing to future directions of research and resources needed to increase the size and quality of data to continue this work.

## Figures and Tables

**Table 1:** Observations registered in database.

Time Stamp	Platform	Sensor	Type of Sensor	Value
0.1 s	*P_1_*	*S_11_*	Hard	(x,y)
0.2 s	*P_2_*	*S_12_*	Hard	(x,y)
0.4 s	*P_1_*	*S_31_*	Soft	ID
0.6 s	*P_3_*	*S_31_*	Hard	(x,y)
0.7 s	*P_3_*	*S_23_*	Soft	ID
0.9 s	*P_1_*	*S_11_*	Hard	(x,y)
…	…	…	…	…

**Table 2:** Set of agricultural products.

Spanish Name	Scientific Name	English Name
Acelga	*Beta vulgaris* var. cicla	Chard
Ajo	*Allium sativum*	Garlic
Arveja	*Pisum sativum*	Vetch
Babaco	*Carica pentagona*	Babaco
Brócoli	*Brassica oleracea italica*	Broccoli
Cebolla blanca	*Allium fistulosum*	White onion
Cebolla paiteña	*Allium fistulosum*	Onion
Choclo	*Zea mays*	Corn
Col	*Brassica oleracea*	Cabbage
Col verde	*Brassica oleracea* var. Sabellica	Green cabbage
Coliflor	*Brassica oleracea* var. Botrytis	Cauliflower
Espinaca	*Spinacia oleracea*	Spinach
Frejol	*Phaseolus vulgaris*	Frejol
Frutilla	*Fragaria*	Strawberry
Habas	*Vicia faba*	Broad beans
Hierbas	*Coriandrum sativum, Petroselinum crispum*	Weeds
Lechuga	*Lactuca sativa*	Lettuce
Melloco	*Ullucus tuberosus*	Melloco
Nabo	*Brassica rapa*	Turnip
Papas	*Solanum tuberosum*	Potatoes
Pepinillo	*Cucumis sativus*	Pickle
Pepino	*Cucumis sativus*	Cucumber
Pimiento	*Capsicum annuum*	Pepper
Rábano	*Raphanus sativus*	Radish
Remolacha	*Beta vulgaris*	Beet
Tomate de árbol	*Solanum betaceum*	Tamarillo
Tomate Riñón	*Solanum lycopersicum*	Tomato
Vainita	*Phaseolus vulgaris L*	Vainita
Zanahoria	*Daucus carota*	Carrot
Zapallo	*Cucurbita maxima*	Squash

**Table 3:** Sales data and spatial location.

FAIR	BROCCOLI	WHITE ONION	TOMATO	TAMARILLO	CARROT	X	Y
Colta	6	60	26	30	30	–78.7238	–1.888
Tisaleo	37.9	36	88.25	185	47.5	–78.6923	–1.40951
Riobamba1	8	9.6	18	0	0	–78.6737	–1.66153
Riobamba2	26	42.5	60	15	17.5	–78.673	–1.66089
Riobamba3	0	10	8	8	5	–78.6687	–1.66025
Riobamba4	2.5	74	60	35	29.5	–78.6588	–1.67626
Cevallos	9	9.6	51	45	20	–78.6566	–1.25714
Riobamba5	7.2	50	30	35	20	–78.6558	–1.6871
Riobamba6	6	65	42	27	29	–78.6538	–1.67599
Riobamba7	2	0	10	0	5	–78.65	–1.67803
Riobamba8	6	26.5	33	0	36	–78.6497	–1.67468
Riobamba9	2.5	0	6.9	0	3	–78.6463	–1.68942
Chambo	21	50	30	20	20	–78.6077	–1.7303
Pillaro	20.1	141.5	92	78	40	–78.551	–1.32836

**Table 4:** Merged file with sales and locations per product.

FAIR	BROCCOLI	WHITE ONION	TOMATO	TAMARILLO	CARROT	X	Y	POPULATION	PCLIMATICO
Colta	6	60	26	30	30	–78.7238	–1.888	44.701	192
Tisaleo	37.9	36	88.25	185	47.5	–78.6923	–1.40951	10.565	192
RIOBAMBA1	8	9.6	18	0	0	–78.6737	–1.66153	193.315	86
RIOBAMBA2	26	42.5	60	15	17.5	–78.673	–1.66089	193.315	86
RIOBAMBA3	0	10	8	8	5	–78.6687	–1.66025	193.315	86
RIOBAMBA4	2.5	74	60	35	29.5	–78.6588	–1.67626	193.315	86
Cevallos	9	9.6	51	45	20	–78.6566	–1.25714	6.8730	70
RIOBAMBA5	7.2	50	30	35	20	–78.6558	–1.68710	193.315	86
RIOBAMBA6	6	65	42	27	29	–78.6538	–1.67599	193.315	86
RIOBAMBA7	2	0	10	0	5	–78.6500	–1.67803	193.315	86
RIOBAMBA8	6	26.5	33	0	36	–78.6497	–1.67468	193.315	86
RIOBAMBA9	2.5	0	6.9	0	3	–78.6463	–1.68942	193.315	86
CHAMBO	21	50	30	20	20	–78.6077	–1.7303	10.541	86
Pillaro	20.1	141.5	92	78	40	–78.551	–1.32836	34.925	70

**Table 5:** Root mean square error (RMSE).

Process	Min	1Q	Median	Mean	3Q	Max	RMSE
IDW	−40.1610	–21.955	1.669	2.259	23.613	44	27.804
Tom (kriging)	–51.2331	–21.1409	4.8034	–0.0194	23.5698	43.9355	29.440
Tom/Population (cK)	–51.2289		4.8066	–0.0945	23.3578	43.9361	29.370
Tom/Prec (cK)	–51.2170	–26.465	4.87	–0.731	23.097	43.944	29.320
Tom/AR (cK)	–34.1243	–11.1405	–3.151	–0.9209	6.2992	29.2215	16.550
Tom/All Var (cK)	–36.8303	–9.4598	1.3216	0.1151	5.5997	34.6041	19.420
